# Prevalence and Risk Factors of Osteoarthritis in Korea: A Cross-Sectional Study

**DOI:** 10.3390/medicina60040665

**Published:** 2024-04-19

**Authors:** Do-Youn Lee

**Affiliations:** College of General Education, Kookmin University, Seoul 02707, Republic of Korea; triptoyoun@kookmin.ac.kr; Tel.: +82-02-910-5540

**Keywords:** osteoarthritis, risk factor, prevalence

## Abstract

*Background and Objectives:* The goal of this study is to determine the prevalence of knee osteoarthritis (OA) and risk factors for the disease in Korean adults over the age of 50, as well as to provide basic data for OA prevention through management. *Materials and Methods:* Based on 2010–2013 data from the Korean National Health and Nutrition Survey, 7962 adults over the age of 50 who participated in radiological tests and health surveys for the diagnosis of osteoarthritis were chosen as participants. *Results:* The risk factors for OA occurrence were investigated using complex sample multiple logistic regression analysis. According to the findings, the prevalence of knee OA in Korea was 33.3% in this study, with the risk of OA being higher in women, the elderly, people with a lower education level, and people with obesity. *Conclusions:* To reduce the incidence of OA, interventions and lifestyle changes are needed to prevent the onset of disease in participants with risk factors for OA, such as older women, low education levels, and obesity.

## 1. Introduction

Knee osteoarthritis (OA) is a disease characterized by gradual degeneration of joint cartilage and pain in the knee, which limits daily activities and reduces functional independence [1,2,3]. OA is common among older adults and has a significant impact on individuals and society [4,5]. The economic burden of knee OA at the national level is increasing as the elderly population grows and life expectancy increases [6]. Furthermore, one previous study warned of the risk due to the high association between osteoarthritis and cardiovascular disease [7].

The prevalence of OA is increasing as the population in industrialized and developing countries ages, as is a rise in risk factors for OA, particularly obesity and a sedentary lifestyle [8,9]. Risk factors for OA can be separated into two categories: person-level factors (age, sex, obesity, and genetics) and joint-level factors (injury, malalignment, and abnormal joint loading), which interact complexly [10]. Knee OA has been linked to higher rates of hospitalization and mortality in long-term care facilities [11], as well as decreased physical activity in older adults due to knee pain symptoms [12,13]. Most of the disease burden associated with knee OA is used in palliative care, such as pain control [14], and to reduce this cost burden, it is critical to identify the disease’s risk factors and shift the paradigm toward prevention rather than treatment.

To lower the prevalence of OA, control of related factors is critical, so it is necessary to analyze the risk factors for OA and investigate their relevance. Previous research on knee OA has found that it is more common in the elderly, obese people, those with high blood pressure, and women, primarily Caucasians [15,16]. Although there are studies on various knee OA risk factors for Caucasians, only a few studies have been conducted in the Asian population, and population-based cohort studies with a large number of participants are somewhat limited. Similar studies on the previous Korean population analyzed risk factors based on quality of life and regional classification, but there were limitations because they only targeted the elderly population over the age of 65 [17,18].

Therefore, this study provides basic data for the development of health management strategies and intervention programs by monitoring the prevalence of knee OA and disease risk factors using national statistical data that are representative and reliable. Therefore, the study’s specific objectives are listed below. First, the prevalence of osteoarthritis is investigated using the Kelgren–Laurence (K–L) grade, an accurate radiological test. Second, differences in socio-demographic characteristics and health behavior types between knee OA and normal groups in the Korean population over 50 years old are compared and analyzed. Third, the goal of this study is to identify the risk factors for knee OA.

## 2. Materials and Methods

**Figure 1:**
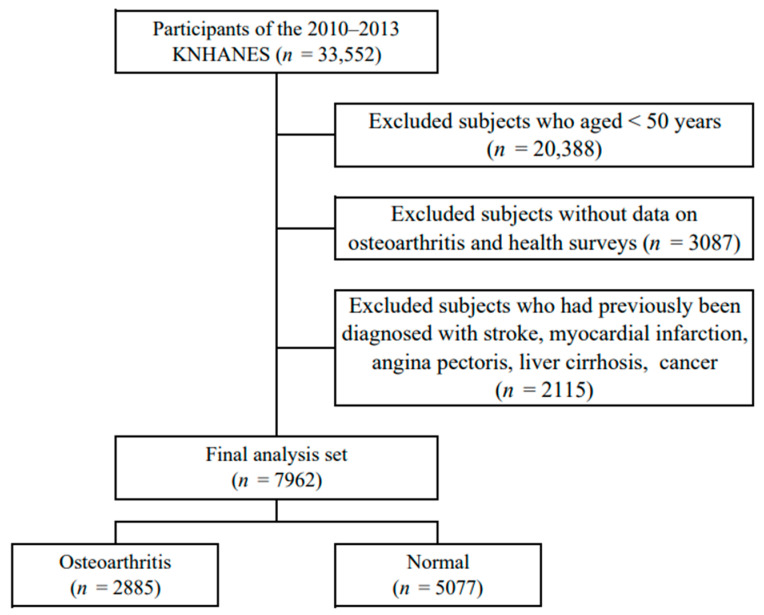
Selection of participants from the Korea National Health and Nutrition Examination Survey 2010–2013.

The Korea National Health and Nutrition Survey (KNHANES), conducted by the Korea Centers for Disease Control and Prevention, provided data for this study from 2010 to 2013. All participants provided their written informed consent for voluntary participation. Adults over the age of 50 completed all health surveys, participated in physical measurement tests such as blood sugar and blood pressure, and underwent all radiological tests for OA diagnosis. Of the 33,552 survey participants, 20,388 were under the age of 50, 3087 did not take the osteoarthritis and health survey, and 2115 had severe diseases such as stroke, myocardial infarction, or angina. Finally, 7962 subjects were selected (Figure 1). This study used data from 2010–2013 from the Korea National Health and Nutrition Survey (KNHANES) conducted by the Korea Centers for Disease Control and Prevention. Among adults over the age of 50, participants completed all health surveys, participated in physical measurement tests such as blood sugar and blood pressure, and underwent all radiological tests for OA diagnosis. Of the 33,552 respondents, 20,388 were under the age of 50, 3087 did not participate in the osteoarthritis and health survey, and 2115 had severe diseases such as stroke, myocardial infarction, or angina. Finally, 7962 participants were chosen (Figure 1).

### 2.1. Demographic Characteristics Factors

Demographic and sociological variables such as sex, age, education level, marital status, and personal income level were recorded. Age was classified as 50 s, 60 s, and 70 s or older. Education levels were classified as low and high based on high school graduation. Marital status was determined by whether or not one lives with the current spouse. For personal income level, the average monthly personal income was divided using the quartile.

### 2.2. Health and Disease-Related Characteristics

The health and disease-related variables measured included height, weight, body mass index (BMI), blood pressure, fasting glucose, triglycerides, high-density lipoprotein cholesterol (HDL-C), waist circumference, smoking and drinking status, and aerobic and resistance exercise. The BMI was calculated by dividing body weight (kg) by the square of height (m^2^). It was classified as underweight, normal, overweight, and obesity. Blood pressure was measured using a mercury spherometer, and after 5 min of stability with a cuff that fits the arm circumference, it was measured three times at 30-s intervals. Hypertension was defined as systolic blood pressure greater than 130 mmHg, diastolic blood pressure greater than 85 mmHg, or current use of antihypertensive medications. Blood tests were taken with an empty stomach for more than 8 h and analyzed within 24 h on a Hitachi Automatic Analyzer 7600 (Hitachi, Tokyo, Japan). Diabetes (hyperglycemia) was defined as fasting glucose levels ≥ 100 mg/dL or taking diabetes medications. Hypertriglyceridemia was classified as triglycerides higher than 150 mg/dL. Low HDL-C was defined as less than 40 mg/dL in men and less than 50 mg/dL in women, while abdominal obesity was defined as more than 90 cm in men and more than 85 cm in women based on waist circumference (WC) [19].

In terms of smoking status, when asked about current smoking status, “daily smoking” and “occasional smoking” were classified as current smoking, “I smoked in the past, but I did not smoke in the present” was classified as past smoking, and “I never smoked” was classified as non-smoking. Current drinking was defined as “more than once a month”, while nondrinking was defined as “less than once a month” and “I did not drink at all in the past year” [19].

Aerobic exercise was calculated using the following walking time: The number of days the subject walked ≥ 10 min at a time for the past week was expressed. Walking was measured by total walking time per week (TWT), which was calculated as follows: TWT = walking days (days/week) × walking minutes (minutes/day) [20]. The frequency of resistance exercise was determined by participants’ responses to the question “How many times do you do resistance exercise (push-ups, sit-ups, lifting dumbbells or barbells) a week?” If there was no resistance exercise at all, it was divided into two groups: medium intensity for 1–3 days of resistance exercise and high intensity for more than 4 days [21].

### 2.3. Radiographic Examination Knee OA Definition

This study diagnosed knee OA via radiological examination. The Kellgren–Lawrence (K–L) grade was used to diagnose radiographic knee OA via knee radiography. The K–L grade, a 5-point scale (0 = normal, 1 = doubtful, 2 = definite, 3 = moderate, and 4 = severe radiographic knee OA), is a standard radiographic assessment of joint degeneration used to diagnose and stage radiographic knee OA. Radiographic knee OA was defined as a K–L grade of ≥2 on knee radiography [22].

### 2.4. Data Analysis

The SPSS 28.0 program (SPSS Inc., Chicago, IL, USA) was used for the data analysis of this study, with a statistical significance level of 0.05. A complex sampling method was used to analyze the data of the KNHANES, which was chosen to ensure that the data used in this study accurately represented the Korean people. The specific analysis methods are specified below.

First, the difference in characteristics between knee OA and the normal group was analyzed using a *t*-test and chi-square test (χ^2^-test). Variance estimation was compared using standard errors (SE). Second, complex sample multiple logistic regression analysis was used to investigate the risk factors for knee OA, with statistics expressed as odds ratios and 95% confidence intervals.

## 3. Results

### 3.1. Subsection Knee OA Prevalence and Demographic Characteristics of Subjects

**Figure 2:**
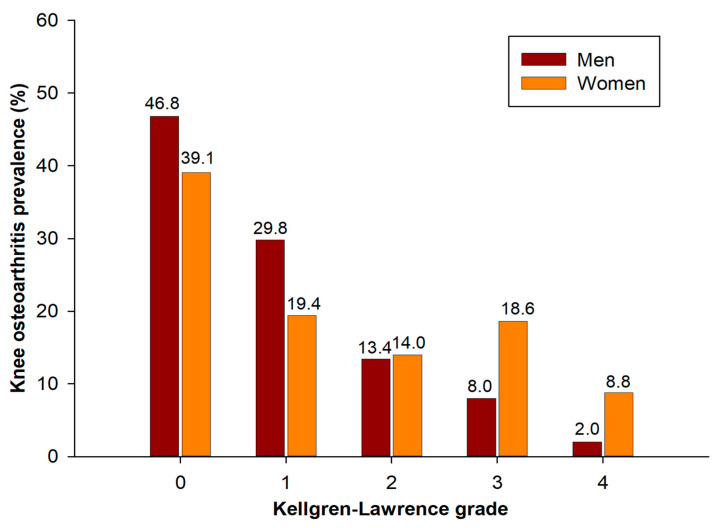
Prevalence of knee osteoarthritis according to Kellgren–Lawrence grade by sex.

The prevalence of knee OA in this study population was 33.3%. In terms of gender prevalence rates, based on K–L grade, 0 was 46.8%, 1 grade was 29.8%, 2 grade was 13.4%, 3 grade was 8.0%, and 4 grade was 2.0%. Women’s rates were 39.1%, 19.4%, 14.0%, 18.6%, and 8.8%, respectively (Figure 2).

The demographic and sociological characteristics of the subjects of this study are shown in Table 1. The demographic and sociological characteristics between the knee OA and the normal group had statistically significant differences in gender, age, education level, and marital status, except for individual income. In men, the prevalence of knee OA was 23.4%, while in women it was significantly higher, at 41.5%. The higher the age group, the lower the level of education, and the greater the risk of knee OA when not living with the current spouse.

### 3.2. Health and Disease-Related Characteristics of the Subject

The health and disease-related characteristics of the participants in this study are highlighted in Table 2. The health and disease-related characteristics of knee OA and the normal group showed statistically significant differences in height, BMI, blood pressure, diabetes, low HDL-C, abdominal obesity, smoking status, drinking status, and aerobic and resistance exercise variables.

### 3.3. Multiple Logistic Regression Analysis for Knee OA Risk Factor

Table 3 shows the risk factors for knee OA. According to simple logistic regression analysis, the factors influencing knee OA were gender, age, education level, marital status, individual income, BMI, hypertension, diabetes, HDL-C, abdominal obesity, smoking and drinking status, and resistance to exercise.

The final multiple logistic regression model’s Nagelkerke R2 = 0.236. In the multiple logistic regression analysis results after controlling for covariates, the risk factors for knee OA were sex, age, education level, and BMI. Women had a higher OR than men, with 1.862 (95% CI 1.487–2.331). In comparison to those in their 50s, OR 2.658 (95% CI 2.290–3.086) was higher in their 60s, and OR 6.384 (95% CI 5.404–7.542) in their 70s. The incidence of knee OA was higher with an OR of 1.760 (95% CI 1.375–2.253) at a low level of education than at a high level. Normal BMI compared to low was OR 0.337 (95% CI 0.214–0.531), overweight was OR 1.591 (95% CI 1.333–1.899), and obesity was OR 3.717 (95% CI 2.614–5.285). This demonstrates that the older and more obese he or she is, the greater the risk of developing knee OA.

## 4. Discussion

This study was conducted to identify the risk factors influencing the prevalence of knee OA and to provide basic data for developing health management strategies and intervention programs to prevent knee OA diseases. According to this study, the prevalence of knee OA in Korean adults over the age of 50 was 33.3%, with 23.4% in men and 41.5% in women.

In this study, the risk factors for knee OA were sex, age, education level, and BMI. The prevalence of knee OA in women was about 1.86 times higher than in men. Women are more likely than men to suffer from knee OA. Although it varies by study, several previous studies found that women had a higher knee OA ratio among the elderly than men, which was consistent with the findings of this study [23,24]. The mechanism of differences in the prevalence of knee OA by sex is still not clearly known. One of the most convincing hypotheses is that estrogen hormone acts on cartilage, leading to an increased risk of developing OA in elderly women due to decreased postmenopausal hormones [25]. These gender differences highlight the importance of targeted assistance and management for knee OA in women.

The risk of developing knee OA increased with age, with a 2.67-fold increase in the 60s and a 6.38-fold increase in the 70s and older compared to the 50s. This is consistent with findings from several previous studies [24,26]. The prevalence of knee OA increases with age, particularly among those over the age of 60 [26]. As the age group increases, the destruction of cartilage is greater than the generation, making it more easily damaged, and because tendon and ligament stiffness occur with the loss of normal skeletal structure, the prevalence of knee OA appears to increase with the age group [24]. There may be several reasons for the increased prevalence of knee OA as age increases. First, the loss of chondrocytes may have occurred during the aging process, and the chondrocytes may have lost their ability to maintain the surrounding extracellular matrix [27]. As a result, synthetic activity may be reduced in aged chondrocytes, and proteoglycans may have become irregular [28]. Second, inflammation caused by aging may have affected the cartilage joint systematically or locally [29]. The higher the inflammation level, the higher the risk of knee arthritis progression [30]. Therefore, it is believed that national health prevention or customized environmental health policy projects will be beneficial to the health management of the older population, who can be considered a vulnerable group for the occurrence of knee OA.

An independent association between educational achievement and knee OA was also found in previous research [31,32]. However, the reason for the link between education level and knee OA prevalence is unclear. The relationship between knee OA prevalence and education level is more likely to be mediated by several indirect factors (e.g., occupational factors, lifestyle, etc.) than a direct relationship [33]. Rural older people have lower overall educational levels and health status, particularly among women [34]. These arguments are supported by previous studies that found a higher prevalence of knee OA in rural areas than in cities [17]. In fact, urban and rural residents have differences in educational levels, resulting in differences in quality of life, which is particularly noticeable in the elderly population [35]. Because education levels are likely to be difficult to intervene in in adulthood, it is critical to identify the factors that can explain these findings and target the potential risk factors that explain the education-related disparities in the prevalence of knee OA.

An increase in BMI is linked to knee OA sensitivity. This association may be closely related to the biomechanical state. Obesity increases the load on the knee joint cartilage, and many of the resulting shocks contribute to the process of knee regression [36,37]. In particular, obesity weakens the quadriceps of the body mass, reducing the primary role of the quadriceps in absorbing shock in the knee. This can increase the stress on the joint cartilage of the knee, resulting in joint regression [38]. Therefore, resistance exercise for weight loss and an active knee joint are required to prevent knee OA.

There were significant differences in simple regression analysis for hypertension, hyperglycemia, smoking, and drinking status, but not in multiple regression analysis with several covariates. Several previous studies also yielded similar results to this study. As in the results of this study, there were no significant differences in the association between several chronic diseases such as hypertension, diabetes, low HDL-cholesterolemia, and hypertriglyceridemia, except obesity based on BMI, in several previous studies [39,40,41]. Furthermore, one previous study found no evidence that smoking, drinking, or exercising increases the risk of OA occurrence [40]. In contrast, another study found a link between male knee OA and the frequency of physical activity [42]. These contradictory results are thought to be due to a difference in subject selection in this study, which was based on an age range of 20–69 years in the previous study.

The study has several limitations. First, this is a cross-sectional study that uses secondary data analysis to confirm the factors that influence the occurrence of knee OA at the time of investigation. Therefore, the causal relationship can only be explained to a certain extent. The causal relationship must be confirmed through cohort studies in the future. Second, there was a draw recall bias because the socio-statistical characteristics of the research population were obtained via questionnaires. However, this technique was most likely eliminated at random and had no influence on the study’s findings. Third, this study contains no data on hormones. Several studies have found that sex hormones, reproductive factors, and hormone supplements contribute to osteoarthritis [43]. Fourth, although various demographic and health-related variables were included in this study, there may be variables that have other effects. Therefore, future research will need to analyze the levels of endogenous hormones as well as hormone supplementation. Despite these limitations, this study is important as primary evidence for health promotion projects targeting knee OA patients.

## 5. Conclusions

This study was conducted to present basic data for the prevention and management of knee OA by identifying the prevalence and risk factors of knee OA in adults over 50 years of age in Korea. In this study, the overall knee OA prevalence was 33.3%, and when classified by sex, men accounted for 23.4% and women accounted for 41.5%. According to the analysis of the factors influencing the occurrence of knee OA, the higher the risk of occurrence in women, the older the patient, the lower the level of education, and the presence of obesity based on BMI. Therefore, to prevent diseases, it is necessary to develop and provide health-related education programs that can be completed by high-BMI and low-educated elderly women.

## Figures and Tables

**Table 1:** Socio-demographic characteristics of subjects according to osteoarthritis.

Factors	Categories	OA (*n* = 2885)		Normal (*n* = 5077)		χ^2^	*p* for Trend
		U/F	W/F	U/F	W/F		
		N or Mean ± SD	%	N or Mean ± SD	%		
Sex	Men	865	23.4	2401	76.6	290.91	<0.001
	Women	2020	41.5	2676	58.5		
Age		65.53 ± 0.23		58.86 ± 0.14			<0.0001
	50–59	625	19.2	2664	80.8	955.73	
							<0.001
	60–69	1025	39.9	1595	60.1		
	≥70	1235	60.6	818	39.4		
Education	Low	2695	35.9	4209	64.1	167.75	<0.001
	High	190	15.8	868	84.2		
Marital status	with	2008	29.3	4287	70.7	221.10	<0.001
	without	877	48.8	790	51.2		
Individual income	Q1 (Lowest)	776	27.5	1178	24.4	12.49	0.056
	Q2	716	25.6	1252	25.1		
	Q3	690	23.8	1334	26.4		
	Q4 (Highest)	703	23.0	1313	24.1		

Data were presented as means ± SE (%).

**Table 2:** Health and disease related characteristics in subjects according to osteoarthritis.

Factors	Categories	OA (*n* = 2885)		Normal (*n* = 5077)		χ^2^	*p*
		U/F	W/F	U/F	W/F		
		N or Mean ± SD	%	N or Mean ± SD	%		
Height (cm)		157.146 ± 0.21		161.349 ± 0.14			<0.0001
Weight (kg)		61.749 ± 0.26		62.224 ± 0.18			0.138
BMI (kg/m^2^)		24.925 ± 0.08		23.83 ± 0.06			<0.0001
	Low	36	1.0	128	2.3	207.4	<0.0001
	Normal	1579	54.3	3384	65.9		
	Overweight	1093	38.0	1469	29.9		
	Obesity	177	6.8	96	1.9		
Blood pressure (mmHg)	Systolic	128.609 ± 0.42		124.344 ± 0.35			<0.0001
	Diastolic	76.764 ± 0.25		78.463 ± 0.21			<0.0001
	Hypertension	1459	49.2	2135	42.1	36.5	<0.001
Fasting glucose (mg/dL)		104.127 ± 0.55		102.99 ± 0.42			0.087
	Diabetes	1276	45.2	2056	41.0	12.78	0.005
TG		146.947 ± 2.15		151.307 ± 2.07			0.141
	High	991	36.8	1790	38.0	1.02	0.391
HDL-cholesterol		48.425 ± 0.29		48.665 ± 0.22			0.492
	Low	1444	50.1	2108	40.5	66.91	<0.001
WC (cm)		85.108 ± 0.24		82.737 ± 0.17			<0.0001
	abdominal obesity	1222	42.4	1379	27.3	183.10	<0.0001
Smoking status	current	330	14.2	937	21.9	189.81	<0.0001
	past	507	17.4	1279	26.0		
	non	2048	68.4	2861	52.2		
Alcohol status	Yes	1575	57.2	3408	70.0	127.12	<0.0001
	No	1310	42.8	1669	30.0		
Aerobic exercise	TWT	260.088 ± 9.75		285.475 ± 7.98			<0.001
Resistance exercise	Never	2410	82.6	3829	74.9	61.47	<0.001
	Mid	228	8.8	663	13.6		
	High	247	8.6	585	11.5		

Data were presented as means ± SE (%). BMI = body mass index; TG = triglyceride; HDL = high density lipoprotein; WC = waist circumference.

**Table 3:** Multiple logistic regression analysis for knee osteoarthritis risk factor.

Variables		Crude		Adjusted	
		OR (95% CI)	*p*	OR (95% CI)	*p*
Sex	Men	1		1	
	Women	2.322 (2.067–2.609)	<0.001	1.862 (1.487–2.331)	<0.001
Age	50–59	1		1	
	60–69	2.795 (2.425–3.22)	<0.001	2.658 (2.290–3.086)	<0.001
	≥70	6.485 (5.588–7.526)	<0.001	6.384 (5.404–7.542)	<0.001
Education	Low	2.980 (2.384–3.725)	<0.001	1.760 (1.375–2.253)	<0.001
	High	1		1	
Marital status	with	1		1	
	without	2.302 (2.0124–2.634)	<0.001	1.122 (0.966–1.304)	0.131
Individual income	Q1 (Lowest)	1.188 (1.007–1.401)	0.042	1.116 (0.935–1.331)	0.223
	Q2	1.075 (0.905–1.276)	0.417	1.006 (0.836–1.212)	0.946
	Q3	0.952 (0.802–1.13)	0.571	0.932 (0.774–1.122)	0.456
	Q4 (Highest)	1		1	
BMI (kg/m^2^)	Low	0.528 (0.333–0.839)	0.007	0.337 (0.214–0.531)	<0.001
	Normal	1		1	
	Overweight	1.539 (1.357–1.744)	<0.001	1.591 (1.333–1.899)	<0.001
	Obesity	4.293 (3.188–5.782)	<0.001	3.717 (2.614–5.285)	<0.001
Blood pressure	Normal	1		1	
	Hypertension	1.335 (1.191–1.495)	<0.001	1.133 (0.995–1.291)	0.06
Fasting glucose (mg/dL)	Normal	1		1	
	Diabetes	1.188 (1.054–1.338)	0.005	1.025 (0.895–1.174)	0.722
TG	Normal	1		1	
	High	0.952 (0.85–1.067)	0.391	1.036 (0.777–1.380)	0.087
HDL-cholesterol	Normal	1		1	
	Low	1.479 (1.317–1.659)	<0.001	1.010 (0.872–1.170)	0.892
WC (cm)	Normal	1		1	
	abdominal obesity	1.957 (1.729–2.215)	<0.001	1.127 (0.933–1.363)	0.214
Smoking status	current	0.496 (0.417–0.59)	<0.001	1.048 (0.820–1.341)	0.706
	past	0.513 (0.445–0.592)	<0.001	0.849 (0.672–1.073)	0.171
	non	1		1	
Alcohol status	Yes	0.575 (0.511–0.647)	<0.001	1.002 (0.876–1.147)	0.973
	No	1		1	
Aerobic exercise	TWT	1.000 (1.000–1.001)	0.053	1.000 (1.000–1.001)	0.325
Resistance exercise	Never	1.479 (1.218–1.795)	<0.001	1.030 (0.825–1.286)	0.794
	Mid	0.870 (0.669–1.131)	0.297	1.036 (0.777–1.380)	0.81
	High	1		1	

## Data Availability

All data were anonymized and can be downloaded from the website (https://knhanes.kdca.go.kr/knhanes, accessed on 12 February 2024).
